# Abrogation of IFN-γ Signaling May not Worsen Sensitivity to PD-1/PD-L1 Blockade

**DOI:** 10.3390/ijms21051806

**Published:** 2020-03-06

**Authors:** Julie Vackova, Adrianna Piatakova, Ingrid Polakova, Michal Smahel

**Affiliations:** 1 Department of Genetics and Microbiology, Faculty of Science, Charles University, BIOCEV, 252 50 Vestec, Czech Republic; julie.vackova@natur.cuni.cz (J.V.); grzelaka@natur.cuni.cz (A.P.); ingrid.polakova@natur.cuni.cz (I.P.); 2 Department of Cell Biology, Faculty of Science, Charles University, BIOCEV, 252 50 Vestec, Czech Republic

**Keywords:** immune checkpoint therapy, cancer, PD-1/PD-L1, IFNGR1, IFN-α, IFN-β, MHC class I

## Abstract

Programmed cell death protein 1 (PD-1)/PD-1 ligand 1 (PD-L1) blockade is a promising therapy for various cancer types, but most patients are still resistant. Therefore, a larger number of predictive biomarkers is necessary. In this study, we assessed whether a loss-of-function mutation of the interferon (IFN)-γ receptor 1 (IFNGR1) in tumor cells can interfere with anti-PD-L1 therapy. For this purpose, we used the mouse oncogenic TC-1 cell line expressing PD-L1 and major histocompatibility complex class I (MHC-I) molecules and its TC-1/A9 clone with reversibly downregulated PD-L1 and MHC-I expression. Using the CRISPR/Cas9 system, we generated cells with deactivated IFNGR1 (TC-1/dIfngr1 and TC-1/A9/dIfngr1). In tumors, IFNGR1 deactivation did not lead to PD-L1 or MHC-I reduction on tumor cells. From potential inducers, mainly IFN-α and IFN-β enhanced PD-L1 and MHC-I expression on TC-1/dIfngr1 and TC-1/A9/dIfngr1 cells in vitro. Neutralization of the IFN-α/IFN-β receptor confirmed the effect of these cytokines in vivo. Combined immunotherapy with PD-L1 blockade and DNA vaccination showed that IFNGR1 deactivation did not reduce tumor sensitivity to anti-PD-L1. Thus, the impairment of IFN-γ signaling may not be sufficient for PD-L1 and MHC-I reduction on tumor cells and resistance to PD-L1 blockade, and thus should not be used as a single predictive marker for anti-PD-1/PD-L1 cancer therapy.

## 1. Introduction

Cancer immunotherapy based on blockade of the programmed cell death protein 1 (PD-1)/PD-1 ligand 1 (PD-L1) pathway with monoclonal antibodies is increasingly applied in clinical practice [1,2,3,4]. Initially, pembrolizumab and nivolumab were approved by the Food and Drug Administration (FDA) in 2014 for the treatment of patients with advanced melanomas [4]. The number of approved antibodies for the blockade of PD-1/PD-L1 signaling has rapidly increased and improved the therapy of different malignant diseases in patients unsuccessfully treated by other methods [1]. Thus far, six antibodies (pembrolizumab, nivolumab, atezolizumab, durvalumab, avelumab, and cemiplimab) have been approved by the FDA [5,6]. Moreover, in 2015, this progress also resulted in the FDA approval of pembrolizumab as a drug for the first-line treatment of metastatic non-small cell lung cancer (NSCLC) with PD-L1 overexpression [7].

However, despite the great success of this immunotherapy, the majority of the treated patients (about 70–80%) did not respond to therapy, and secondary resistance was recorded in some patients, for instance approximately 25% of melanoma patients [8]. Therefore, reliable predictive biomarkers are required to select suitable patients for treatment to avoid unnecessary burden and reduce high expenses. Unraveling the mechanisms of successful PD-1/PD-L1 blockade and resistance to this therapy contributes to the identification of such biomarkers [9].

Genetic analyses have suggested a relationship between treatment failure and impairment of interferon (IFN)-γ signaling in both primary [10] and secondary resistance to PD-1/PD-L1 blockade [11]. Sample analysis of four patients with melanoma who acquired resistance to the PD-1 blockade revealed mutations in genes encoding the Janus-associated kinase (JAK)-1 or JAK-2 in two patients. These mutations led to insensitivity to IFN-γ treatment, which is associated with the downregulation of major histocompatibility complex class I (MHC-I) expression. Moreover, in the third patient, a mutation in the β-2-microglobulin gene resulted in a complete loss of surface MHC-I expression [11]. 

Defects in responsiveness to IFN-γ stimulation were also associated with the primary resistance to blockade of another immune checkpoint, the cytotoxic T-lymphocyte-associated antigen 4 (CTLA-4) [12]. Therefore, IFN-γ signaling may be considered as a predictive biomarker for cancer immunotherapy with immune checkpoint inhibitors [13]. Somatic mutations in the *JAK1* and *JAK2* genes were identified in various types of human malignancies with a range of 6%–12% and 5%–17%, respectively. As these mutations can be responsible for the lack of acquired PD-L1 expression, they might predict patients who are unlikely to benefit from the anti-PD-1/PD-L1 therapy [10].

In our study, we derived mouse tumor cell lines unresponsive to IFN-γ stimulation and analyzed their response to treatment with PD-L1-blocking antibody. Tumors induced by these cells were sensitive to anti-PD-L1 and acquired PD-L1 expression in vivo. This finding suggests that the exclusive abrogation of IFN-γ signaling in tumor cells is not sufficient for an escape from anti-PD-L1 treatment and should not be a reason for the exclusion of patients from this therapy.

## 2. Results

### 2.1. Characterization of TC-1 or TC-1/A9 Cell Lines with IFNGR1 or PD-L1 Deactivation

**Figure 1:**
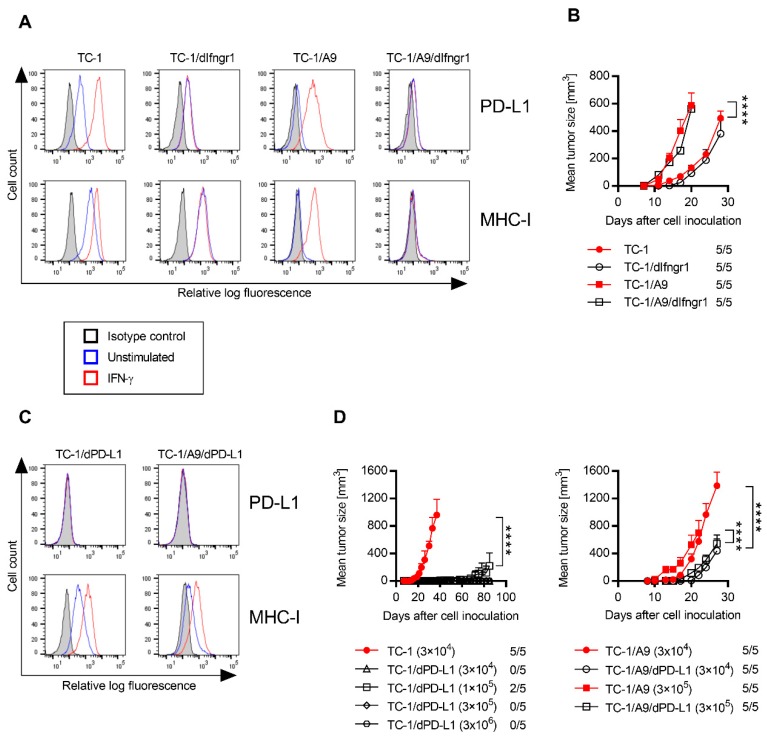
Characterization of the derived cell lines. Surface programmed cell death protein 1 (PD-1) ligand 1 (PD-L1) and major histocompatibility complex class I (MHC-I) expression on unstimulated and stimulated (200 IU/mL interferon (IFN)-γ for 1 day) cells were analyzed by flow cytometry in TC-1, TC-1 clone with a deactivated IFN-γ receptor 1 (IFNGR1; TC-1/dIfngr1), TC-1/A9, and TC-1/A9/dIfngr1 cell lines (**A**) and TC-1/dPD-L1 and TC-1/A9/dPD-L1 cell lines (**C**). Cells were incubated with specific antibodies or isotype control antibodies. (**B**) Oncogenicity of TC-1, TC-1/dIfngr1, TC-1/A9, and TC-1/A9/dIfngr1 cell lines was compared after subcutaneous (s.c.) administration of 3 × 10^4^ cells to C57BL/6 mice (*n* = 5). (**D**) For the evaluation of oncogenicity of cell lines with deactivated PD-L1, various cell doses were s.c. injected. The ratio of mice with a tumor to the total number of mice in the group is shown. Bars ± SEM; **** *p* < 0.0001.

In order to assess whether tumors induced by IFN-γ non-responsive tumor cells may be sensitive to PD-1/PD-L1 blockade and simultaneously enhance the efficacy of immunotherapy of tumors induced by such cells, we prepared TC-1 and TC-1/A9 clones with a deactivated IFN-**γ** receptor. In these cells, we determined the PD-L1 and MHC-I surface expression by flow cytometry (Figure 1A). Although TC-1 cells and TC-1 clone with a deactivated IFN-γ receptor 1 (IFNGR1; TC-1/dIfngr1) markedly expressed PD-L1 and MHC-I molecules, on TC-1/A9 cells and the respective clone with deactivated IFNGR1 (TC-1/A9/dIfngr1), PD-L1 and MHC-I expression were downregulated. After incubation with IFN-γ, PD-L1 and MHC-I expression were increased in TC-1 and TC-1/A9 cells, but TC-1/dIfngr1 and TC-1/A9/dIfngr1 clones did not respond to stimulation, which suggests successful IFNGR1 deactivation. Oncogenicity of the modified clones was similar to that of the parental cells, and TC-1/A9-induced tumors grew significantly faster than TC-1-induced tumors (Figure 1B).

To evaluate the impact of PD-L1 molecules expressed by TC-1 and TC-1/A9 cells on the protection against immune system attack, we generated cellular clones with deactivated PD-L1–TC-1/dPD-L1 and TC-1/A9/dPD-L1, respectively. As assessed by flow cytometry (Figure 1C), both clones remained PD-L1 negative after IFN-γ stimulation. The MHC-I expression was not markedly altered on unstimulated TC-1/dPD-L1 cells, but it was slightly increased on unstimulated TC-1/A9/dPD-L1 cells in comparison with the TC-1/A9 cells. This expression was further enhanced after IFN-γ treatment on both cell lines.

Oncogenicity of the TC-1/dPD-L1 and TC-1/A9/dPD-L1 cells was decreased in comparison with the parental cell lines (Figure 1D). This effect was particularly decisive for the TC-1/dPD-L1 cells that did not form tumors for the doses 3 × 10^4^, 3 × 10^5^, and 3 × 10^6^ and only generated tumors after the injection of 1 × 10^5^ cells in two out of five mice. The TC-1/A9/dPD-L1 cells formed tumors in all mice injected with both 3 × 10^4^ and 3 × 10^5^ cells, but their growth was significantly reduced in comparison with TC-1/A9-induced tumors. Thus, PD-L1 expressed on the TC-1 and TC-1/A9 cells plays an important role in the suppression of anti-tumor immunity. This effect is much more evident for the TC-1 cell line.

### 2.2. Mechanisms Contributing to Anti-Tumor Immunity

**Figure 2:**
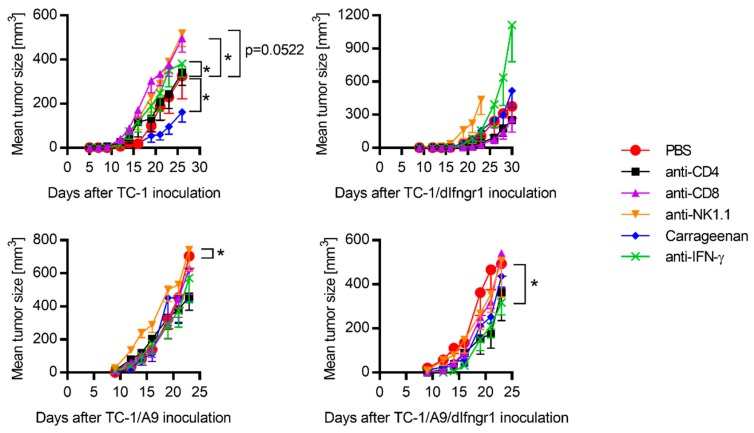
Mechanisms contributing to anti-tumor immunity. C57BL/6 mice (*n* = 5) were s.c. injected with 3 × 10^4^ tumor cells and treated with anti-cluster of differentiation (CD)4, anti-CD8, anti-natural killer (NK)1.1, and anti- interferon (IFN)-γ antibodies or carrageenan. Phosphate-buffered saline (PBS) was injected as a negative control. Bars ± SEM; * *p* < 0.05.

To analyze the effect of IFNGR1 deactivation in tumor cells on a pro-/anti-oncogenic role of immune cells, we depleted cluster of differentiation (CD)4^+^, CD8^+^, or natural killer (NK)1.1^+^ cells, or macrophages in mice bearing tumors with functional or deactivated IFNGR1 (Figure 2). We also neutralized IFN-γ to evaluate its influence on the oncogenicity of tumors induced by cells non-sensitive to this cytokine. Although the depletion of CD8^+^ or NK1.1^+^ cells and IFN-γ neutralization enhanced TC-1-induced tumor growth, depletion of macrophages inhibited the growth of these tumors. For TC-1/dIfngr1-induced tumors, depletion of NK1.1^+^ cells and IFN-γ neutralization had similar effects, but the impact of CD8^+^-cell or macrophage depletion was not preserved. In mice with TC-1/A9 tumors, depletion of NK1.1^+^ cells resulted in significant enhancement of tumor growth. On the contrary, depletion of any cell type did not have a significant influence on the growth of TC-1/A9/dIfngr1-induced tumors. However, IFN-γ neutralization significantly reduced the growth of these tumors.

### 2.3. PD-L1 and MHC-I Surface Expression on Tumor Cells

**Figure 3:**
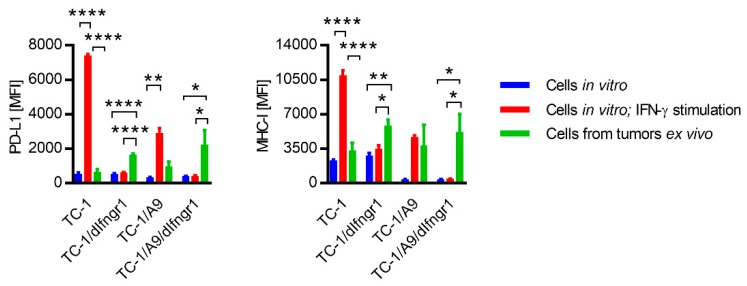
Ex vivo analysis of PD-L1 and MHC-I surface expression by tumor cells. Cells (3 × 10^4^) were s.c. injected into C57BL/6 mice (*n* = 3) to form tumors. Cells harvested from tumors were stained by specific antibodies and analyzed by flow cytometry. CD45^-^ tumor cells were compared with cell lines. In vitro cultured cell lines were untreated or stimulated for 1 day with 200 IU/mL IFN-γ. Median fluorescence intensity, MFI; columns, means of three samples; Bars, ± SEM. * *p* < 0.05, ** *p* < 0.01, **** *p* < 0.0001.

We determined the intensity of PD-L1 and MHC-I expression on tumor cells derived from the TC-1, TC-1/dIfngr1, TC-1/A9, and TC-1/A9/dIfngr1 tumors ex vivo, in order to analyze the impact of the tumor microenvironment on both molecules (Figure 3). Except for the TC-1-induced tumors, the ex vivo expression was markedly increased in comparison with in vitro cultured unstimulated cells.

Furthermore, this expression on cells isolated from TC-1/dIfngr1 and TC-1/A9/dIfngr1 tumors was even slightly higher than on cells from TC-1 and TC-1/A9 tumors. Moreover, for TC-1/A9- and for TC-1/A9/Ifngr1-induced tumors, the expression of MHC-I in particular was comparable to that observed on TC-1/A9 cells stimulated with IFN-γ in vitro. These results showed that deactivation of the IFN-γ receptor did not reduce the level of PD-L1 and MHC-I expression in tumor cells in vivo. It suggests that other factors, besides IFN-γ, contributed to the induction of PD-L1 and MHC-I expression in tumor cells.

### 2.4. Cytokines Inducing PD-L1 and MHC-I Expression on Tumor Cells

**Figure 4:**
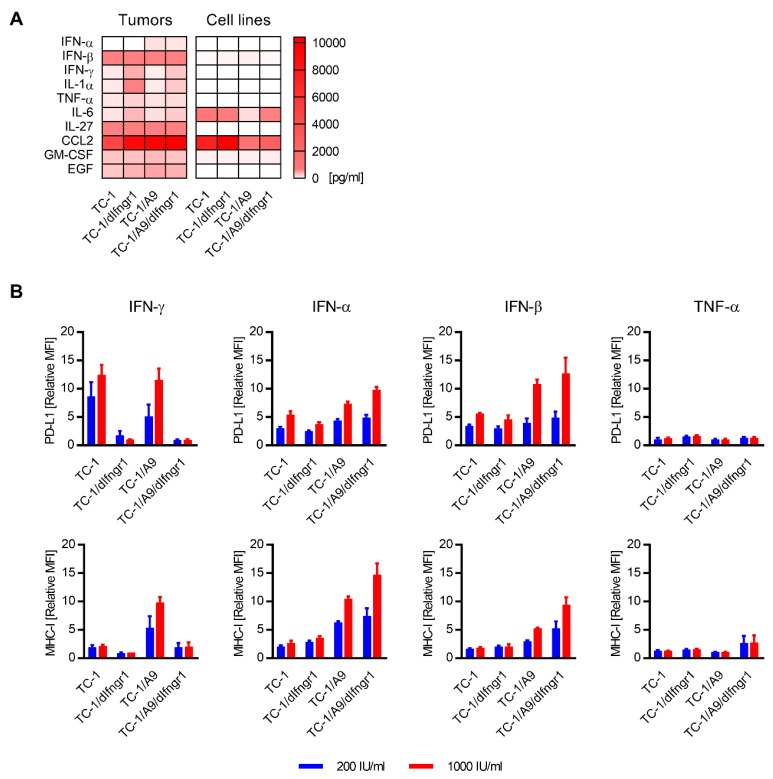
The effect of cytokines on PD-L1 and MHC-I surface expression. (**A**) The quantification of cytokines that can enhance PD-L1 and/or MHC-I expression in tumors and supernatants of cells cultured in vitro. (**B**) The effect of IFN-γ, IFN-α, IFN-β, or TNF-α on PD-L1 and MHC-I expression in vitro. Median fluorescence intensity, MFI. Relative MFI was calculated as MFI_stimulated cells_/MFI_unstimulated cells_. Columns, means of three samples; bars, ± SEM.

Consequently, we analyzed the occurrence of presumed PD-L1 and/or MHC-I inducers (IFN-γ, IFN-α, IFN-β, interleukin (IL)-1α, IL-6, IL-27, tumor necrosis factor (TNF)-α, chemokine CCL-2, granulocyte-macrophage colony stimulating factor (GM-CSF), and epidermal growth factor (EGF)) in tumors induced by TC-1, TC-1/dIfngr1, TC-1/A9, and TC-1/A9/dIfngr1 cells and also in cell culture supernatants in vitro (Figure 4A). We found almost all these cytokines in tumors. The only exception was the absence of IFN-α in TC-1- and TC-1/dIfngr1-induced tumors. All in vitro cultured cell lines produced CCL-2 and IL-6. As the surface expression of PD-L1 and MHC-I is low on the unstimulated TC-1/A9 and TC-1/A9/dIfngr1 cells, CCL-2 and IL-6 should not induce PD-L1 and MHC-I expression. Therefore, we excluded these two cytokines from further analysis and evaluated the effect of the remaining cytokines on PD-L1 and MHC-I expression in vitro (Figure 4B). All cell lines were sensitive to type I IFNs (IFN-α and IFN-β) and cultivation of cells in the presence of these cytokines induced PD-L1 and MHC-I expression. Relative increase of PD-L1 on TC-1 cells, unlike MHC-I, was substantial after incubation with IFN-γ. Both PD-L1 and MHC-I were increased on TC-1/A9 cells after treatment with IFN-γ and type I IFNs. TC-1/A9/dIfngr1 cells were considerably more sensitive to type I IFNs than TC-1/A9. TNF-α had minor effect on all cell lines and negligibly elevated MHC-I expression only on TC-1/A9/dIfngr1 cells. None of the cell lines responded to other tested cytokines IL-1α, IL-27, GM-CSF, and EGF (data not shown).

### 2.5. PD-L1 and MHC-I Expression ex Vivo after IFN-α and IFN-β Signaling Blockade

**Figure 5:**
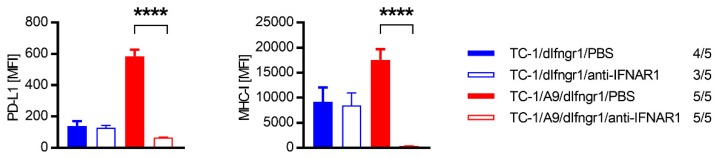
The role of IFN-α and IFN-β in TC-1/dIfngr1- and TC-1/A9/dIfngr1-induced tumors. Cells (3 × 10^4^) were s.c. injected into C57BL/6 mice (*n* = 5) to form tumors. Mice were treated with anti-IFNAR1 antibody, or PBS was injected as a negative control. Cells isolated from tumors were stained by specific antibodies and analyzed by flow cytometry. The ratio of mice with tumor to the total number of mice in the group is shown. Median fluorescence intensity, MFI; columns, means of analyzed samples; bars, ± SEM; **** *p* < 0.0001.

To elucidate the role of IFN-α and IFN-β in tumors induced by cells insensitive to IFN-γ, we applied the antibody against the receptor subunit shared by both interferons—IFN-α receptor 1 (IFNAR1)—and compared PD-L1 and MHC-I expression in tumors from mice treated with phosphate-buffered saline (PBS) or anti-IFNAR1 (Figure 5). Although the expression of these molecules was not changed on TC-1/dIfngr1 cells isolated from tumors, it was notably reduced on TC-1/A9/dIfngr1 cells after anti-IFNAR1 treatment.

### 2.6. Sensitivity to Combined Immunotherapy

**Figure 6:**
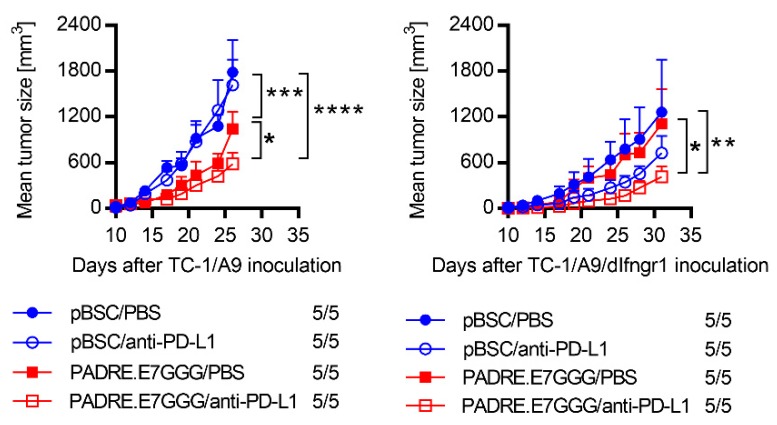
The sensitivity of TC-1/A9 and TC-1/A9/dIfngr1 cells to anti-PD-L1 therapy combined with DNA immunization. Cells (3 × 10^4^) were s.c. injected to C57BL/6 mice (*n* = 5). Animals were treated with anti-PD-L1 antibody and/or the Pan DR epitope (PADRE).E7GGG DNA vaccine delivered by a gene gun. Control mice received PBS and/or pBSC. The ratio of mice with tumor to the total number of mice in the group is shown. Bars ± SEM; * *p* < 0.05, ** *p* < 0.01, *** *p* < 0.001, **** *p* < 0.0001.

As IFN-α and IFN-β induced PD-L1 and MHC-I expression on tumor cells in TC-1/A9/dIfngr1-originated tumors, we examined the sensitivity of these tumors to anti-PD-L1 therapy (Figure 6). We also included DNA vaccination against the human papillomavirus type 16 (HPV16) E7 oncoprotein to enhance the immune response against the tumor-specific antigen. In both TC-1/A9- and TC-1/A9/dIfngr1-induced tumors, the combined immunotherapy significantly reduced tumor growth and anti-PD-L1 treatment significantly supported the effect of DNA immunization.

## 3. Discussion

Although immune checkpoint blockade offers new promising possibilities in cancer treatment, the number of non-responding patients is still high. Therefore, finding predictive markers is necessary to identify potential responders. PD-L1 expression in tumors was used as the first predictive marker for immune checkpoint blockade. Thus far, the FDA has registered four different immunohistochemical assays to assess PD-L1 expression in tumors. However, PD-L1 expression evaluated as a single biomarker appeared predictive in only 28.9% of FDA approvals of immune checkpoint inhibitors [14]. Among the many different markers currently being studied, tumor sensitivity to IFN-γ is considered advantageous for immune checkpoint therapy. In tumors of non-responders, reduced sensitivity to IFN-γ and defective MHC-I expression was found [11]. However, this work did not focus on mutations in tumors of responders to the therapy.

In this study, we tested the sensitivity of tumors induced by cells with functionally deactivated IFN-γ receptor to PD-L1 blockade. To address this question, we used TC-1 [15] and TC-1/A9 cell lines [16]. Although TC-1 cells markedly express PD-L1 and MHC-I molecules, this expression is reversibly downregulated on TC-1/A9 cells and can be induced by cytokines such as IFN-γ. To evaluate how the PD-L1 expression on TC-1 and TC-1/A9 cell lines influences oncogenicity and potential sensitivity to PD-L1 blockade, we prepared TC-1/dPD-L1 and TC-1/A9/dPD-L1 cells with deactivated PD-L1, respectively. PD-L1 on TC-1 cells strongly promoted tumor formation because, after PD-L1 deactivation, most mice did not form tumors from TC-1/dPD-L1 cells administered at different doses. The role of PD-L1 in tumor protection was lower for TC-1/A9 cells, as PD-L1 deactivation on these cells only reduced tumor growth. Because MHC-I expression on TC-1/A9/dPD-L1 cells was downregulated, these cells can be less sensitive to CD8^+^ T cell cytotoxicity than TC-1/dPD-L1 cells, and the protective role of PD-L1 on TC-1/A9/dPD-L1 cells can thus be lower. Previous studies in various mouse tumor models showed that PD-L1 expression on both tumor and host cells can contribute to tumor escape [17,18,19,20,21]. The relative contribution of PD-L1 molecules expressed on tumor cells versus non-tumor cells to tumor protection was dependent on the used model.

After IFNGR1 deactivation, we evaluated the effect of this modification on tumor growth and anti-tumor immunity. Oncogenicity of the TC-1/dIfngr1 and TC-1/A9/dIfngr1 clones remained similar to that of the parental cell lines. It resembles the unchanged growth of mice tumors induced by intradermal injection of B16 cells with knockdown of the *Ifngr1* gene [12]. On the contrary, mouse ovarian cancer HM1 cell-induced tumors with *Ifngr1* knockdown grew more slowly than control tumors [22]. However, these tumors were induced in the peritoneal cavity.

The role of IFN-γ in tumor development is context-dependent [23]. On the one hand, IFN-γ promotes tumor rejection by enhancement of antigen presentation (including MHC-I upregulation) and cytotoxicity of immune cells [24], and on the other hand, a selective pressure of prolonged exposure to IFN-γ can result in downregulation of antigen presentation [25]. Moreover, IFN-γ stimulates PD-L1 and many other molecules inhibiting the anti-cancer response [26,27] and activates some immunosuppressive cells [28]. We also found different roles of IFN-γ in this study. Although in TC-1- and TC-1/dIfngr1-induced tumors, IFN-γ had an anti-tumor effect and in TC-1/A9-induced tumors, it did not markedly affect tumor growth, this cytokine supported the growth of TC-1/A9/dIfngr1-induced tumors, which was mediated by non-tumor cells sensitive to IFN-γ. IFNGR1 deactivation influenced the effect of certain immune cells on tumor development, particularly on TC-1 versus TC-1/dIfngr1 tumors, where IFNGR1 deactivation eliminated the anti-tumor effect of CD8^+^ cells and pro-tumor effect of macrophages. Mechanisms involved in these changes are not currently clear.

As deactivation of IFNGR1 did not lead to the downregulation of the PD-L1 and MHC-I molecules on TC-1/dIfngr1 and TC-1/A9/dIfngr1 cells in vivo compared to TC-1 and TC-1/A9 cells, respectively, we hypothesized that besides IFN-γ, other cytokines induced PD-L1 and MHC-I expression. Several studies have indicated the involvement of different factors (IFN-α, IFN-β, IL-27, EGF, TNF-α, IL-6, GM-CSF, IL-1α, and CCL2 combined with lipocalin 2) in PD-L1 and MHC-I stimulation or stabilization [27,29,30,31,32,33,34,35,36,37,38,39]. In this study, type I IFNs (IFN-α and IFN-β) were the main inducers of PD-L1 and MHC-I expression. We found both type I IFNs in TC-1/A9/dIfngr1-induced tumors but only IFN-β in TC-1/dIfngr1-induced tumors. This observation corresponds to the expression of PD-L1 and MHC-I after IFNAR1 neutralization, which significantly downregulated this expression in TC-1/A9/dIfngr1- but not in TC-1/dIfngr1-induced tumors. It implies that from the type I IFNs, IFN-α mainly contributed to PD-L1 and MHC-I stimulation on tumor cells in the tumor microenvironment. In general, type I IFNs are important inducers of an immune response against tumors [40]. As suggested by the experimental treatment of tumors with the IFN-α-anti-PD-L1 fusion protein, they can also enhance the efficacy of PD-L1 blockade [41].

As we found the in vivo effect of IFN-α on PD-L1 and MHC-I expression for TC-1/A9/dIfngr1-induced tumors, we tested PD-L1 blockade on these tumors. Due to poor immunogenicity of tumors with MHC-I downregulation, we combined PD-L1 blockade with DNA vaccination against the HPV16 E7 oncoprotein representing a tumor-specific antigen. After combined immunotherapy, the PD-L1 blockade significantly contributed to the inhibition of both TC-1/A9 and TC-1/A9/dIfngr1 tumor growth. Therefore, we searched whether mutations in IFN-γ signaling in human tumors occur in responders to PD-1/PD-L1 blockade. In five independent studies on different tumor types with 115 patients defined as responders to PD-1/PD-L1 blockade (i.e., patients with durable clinical benefit, complete response, or partial response according to the response evaluation criteria in solid tumors (RECIST) v1.1), seven patients (6.1%) had a missense or nonsense mutation in IFN-γ signaling: one patient in *Ifngr1* and *JAK-2*, one in *JAK-1*, and five in *JAK-2* [42,43,44,45,46]. Out of 250 non-responders, missense, nonsense, or splice-site mutations in IFN-γ signaling were found in only 11 patients (4.4%): three in *Ifngr1*, one in *Ifngr2*, four in *JAK-1*, and three in *JAK-2* [42,44,45,47].

The sensitivity of tumor cells to IFN-γ can play a critical role in the response to PD-1/PD-L1 blockade, which was demonstrated on lung cancer cell lines [48]. However, our data provide evidence that deactivated IFN-γ signaling may not be sufficient for PD-L1 and MHC-I reduction on tumor cells and resistance to PD-L1 blockade. We demonstrate in our TC-1/A9 tumor model that PD-L1 and MHC-I induction can be performed by other cytokines, such as IFN-α and/or IFN-β. Mutations in IFN-γ signaling in tumors of patients that responded to PD-1/PD-L1 blockade suggest that similar conditions can be found in human tumors. In cases when the resistance to PD-1/PD-L1 blockade was associated with the abrogation of IFN-γ signaling, other mutation(s) might be needed. As the tumor microenvironment is a complex system, the selection of patients suitable for PD-1/PD-L1 blockade should be conducted on the basis of a broad range of predictive markers that can include sensitivity to both IFN-γ and type I IFN signaling. The only impairment in IFN-γ pathway should not be a contraindication to anti-PD-1/PD-L1 therapy.

## 4. Materials and Methods

### 4.1. Mice

Six- to eight-week-old female C57BL/6N mice (Charles River, Sulzfeld, Germany) were used in the experiments. Animals were maintained under standard conditions and in accordance with the guidelines for the proper treatment of laboratory animals at the animal facility of the Czech Center of Phenogenomics (BIOCEV, Vestec, Czech Republic). All animal experimental procedures were carried out in compliance with Directive 2010/63/EU and animal protocols were approved by the Sectoral Expert Committee of the Czech Academy of Sciences for Approval of Projects of Experiments on Animals (reference number 46/2016, 16 May 2016).

### 4.2. Cell Lines and Culture Conditions

TC-1 cell line (Cellosaurus ID: CVCL_4699; provided by T.-C. Wu, John Hopkins University, Baltimore, MD, USA) was prepared by the transformation of C57BL/6 mouse primary lung cells with the HPV16 *E6/E7* oncogenes and human activated *H-ras* [15]. From a TC-1-induced tumor that developed in a mouse preimmunized against the E7 antigen, the TC-1/A9 clone was selected on the basis of a reduced surface expression of MHC-I molecules. This MHC-I downregulation is reversible and can be restored with the IFN-γ treatment [16].

To abrogate the function of the IFN-γ receptor in TC-1 and TC-1/A9 cells, the *Ifngr1* gene (NCBI reference sequence NM_010511.2) was deactivated with the CRISPR/Cas9 system. Two target sites (ATTAGAACATTCGTCGGTAC in exon 2 and CGACCGTATGTTTCGTATGT in exon 5) were designed using the CRISPR Design Tool (http://crispr.mit.edu/) and cloned into the GeneArt CRISPR Nuclease Vector carrying the human CD4 gene (Life Technologies, Carlsbad, CA, USA). The constructed plasmids verified by sequencing were transfected by Lipofectamine 2000 (Thermo Fisher Scientific, Waltham, MA, USA) into TC-1 and TC-1/A9 cells, and three days after transfection, the successfully transfected cells were marked with magnetically labeled antibody against CD4 (Thermo Fisher Scientific), enriched by magnetic isolation, and cloned by serial dilution. The function of the IFNGR1 in clonal cell lines was tested after 40-hour incubation with 200 IU/mL IFN-γ PeproTech, Rocky Hill, NJ, USA) by flow cytometric analysis of MHC-I expression (see above). Next, the Pdcd-1L1 Double Nickase Plasmid (m) kit (sc-425636-NIC, Santa Cruz Biotechnology, Dallas, TX, USA) was used for deactivation of PD-L1 in TC-1 and TC-1/A9 cells. The transfected cells were preselected by puromycin (6 μg/mL added to the culture media 2 days after transfection) for 4 days. TC-1/dPD-L1 and TC-1/A9/dPD-L1 clone selection were performed by single-cell sorting into a 96-well plate by a FACSAria Fusion flow cytometer (BD Biosciences, Franklin Lakes, NJ, USA). Cells were stained with anti-PD-L1-PE (phycoerythrin) antibody (clone 10F.9G2; BioLegend, San Diego, CA, USA) and negatively selected.

All cells were grown in high-glucose Dulbecco’s modified Eagle’s medium (DMEM; Sigma-Aldrich, Merck KGaA, Darmstadt, Germany) supplemented with 10% fetal calf serum (FCS; Biosera, Nuaille, France), 100 IU/mL penicillin, and 100 μg/mL streptomycin (Biosera). Cells were passaged twice a week with 0.05% trypsin containing 0.02% ethylenediaminetetraacetic acid (EDTA; Sigma-Aldrich, Merck KGaA) in PBS (Sigma-Aldrich, Merck KGaA). In vitro cell stimulations were performed in 35 mm cell culture dishes. Cytokines IFN-γ (200 or 1000 IU/mL; PeproTech), IFN-α (200 or 1000 IU/mL; BioLegend), IFN-β (200 or 1000 IU/mL; R&D Systems, Minneapolis, MN, USA), and TNF-α (200 or 1000 IU/mL; PeproTech), IL-27 (5 IU/mL; BioLegend), IL-1α (1000 IU/mL; BioLegend), EGF (10 IU/mL; BioLegend), or GM-CSF (200 IU/mL; BioLegend) were added to 2 mL of culture media for 1 day.

### 4.3. Plasmids

Plasmids pBSC [49] or pBSC/Pan DR epitope (PADRE).E7GGG [50] were used for immunization. The *PADRE.E7GGG* fusion gene consists of the mutated HPV16 *E7* gene (*E7GGG*) containing three point mutations resulting in substitutions D21G, C24G, and E26G in the Rb-binding site [49] and the universal helper Pan DR epitope (PADRE) designed in silico [51].

The plasmids were transformed into the competent *E. coli* XL-1 blue strain, cultured in Luria Broth Medium (Sigma-Aldrich, Merck KGaA) with 100 μg/mL of ampicillin (Duchefa Biochemie, Haarlem, The Netherlands), and purified with the Qiagen Plasmid Maxi Kit (Qiagen, Hilden, Germany).

### 4.4. Preparation of Gene Gun Cartridges

Plasmid DNA was coated onto 1 μm gold particles (Bio-Rad, Hercules, CA, USA) by the procedure recommended by the producer of gold particles. Each cartridge contained 1 μg DNA coated onto 0.5 mg of gold particles.

### 4.5. Animal Experiments

Immunocompetent C57BL/6N mice (five per group) were challenged with tumor cells suspended in 150 μL PBS by subcutaneous (s.c.) injection into the back of animals, under anesthesia with ketamine (100 mg/kg; Bioveta, Ivanovice na Hane, Czech Republic) and xylazine (16 mg/kg; Bioveta). Tumor growth was monitored three times a week, and tumor volume was calculated using the formula (π/6) (a × b × c) where a, b, and c are the length, width, and height of the tumor, respectively.

Mice were immunized with the pBSC/PADRE.E7GGG plasmid by a gene gun (Bio-Rad, Hercules, CA, USA) on days 3, 6, and 10 after TC-1/A9 or TC-1/A9/dIfngr1 inoculation. DNA vaccination was performed at a discharge pressure of 400 psi into the shaven skin of the abdomen. Each immunization consisted of two shots delivering together 2 μg of plasmid DNA. The empty pBSC plasmid was used as a negative control.

In in vivo depletion experiments, the following doses of monoclonal antibodies (Bio X Cell, West Lebanon, NH, USA) in 200 μL PBS were intraperitoneally (i.p.) injected: 100 μg of anti-CD4 (clone GK1.5), 100 μg of anti-CD8 (clone 2.43), and 100 μg of anti-NK1.1 (clone PK136). The efficacy of depletions was verified by staining of splenocytes. To deplete macrophages, 1 mg of carrageenan IV (Sigma-Aldrich, Merck KGaA) dissolved in 200 µl PBS was inoculated i.p. neutralization of the IFNAR1 was achieved with 200 μg of anti-IFNAR1 (clone MAR1-5A3) and IFN-γ with 300 μg of anti-IFN-γ (clone P4-6A2) in 200 μL PBS per mouse. Depletions and neutralizations were performed two days before tumor-cell injection and then twice a week (or once a week in the case of anti-IFN-γ) after tumor cell inoculation.

### 4.6. Tumor Cell Preparation

For flow cytometry analysis, tumors with a volume of about 100 mm^3^ were removed from the animals. The tumor tissue was rinsed with PBS, cut to pieces, and treated with 1 mg/mL collagenase NB 8 (SERVA, Heidelberg, Germany) and 100 µg/mL DNase I (Roche, Basel, Switzerland) in Roswell Park Memorial Institute (RPMI) 1640 medium (Sigma-Aldrich, Merck KGaK) without FCS. To achieve a single cell suspension, the treated tissue was mechanically and also enzymatically dissociated at 37 °C using predefined programs on a gentleMACS Octo Dissociator (Miltenyi Biotec, Bergisch Gladbach, Germany). The obtained cell suspension was filtered through a 70 µm cell strainer and washed with RPMI medium. Erythrocytes were removed with an ammonium-chloride-potassium (ACK) lysing buffer (0.15 M NH_4_Cl, 10 mM KHCO_3_, 1 mM EDTA, pH 7.2–7.4). Cell suspension was filtered through a 42 µm mesh.

### 4.7. Tumor Lysate Preparation

To measure cytokine concentration, tumors with an average weight of 700 mg were immersed in liquid nitrogen, immediately thawed, and cut into pieces. The tissue was homogenized in extraction buffer (100 mM Tris, pH 7.4, 150 mM NaCl, 1 mM ethylene glycol tetraacetic acid (EGTA), 1mM EDTA, 1% Triton X-100, 1mM phenylmethylsulfonyl fluoride, and Pierce protease inhibitor (Thermo Fisher Scientific)) at a ratio of 300 mg of the tissue to 1 mL of buffer at 4 °C. Samples were constantly agitated in a gentleMACS Octo Dissociator at 4 °C for 2 hours and then centrifuged at 10,000× *g* at 4 °C for 20 min. The supernatant was aliquoted and stored at −80 °C.

### 4.8. Flow Cytometry

Single-cell suspensions of the cell lines were incubated with Fixable Viability Dye (eFluor 455UV; eBioscience) to label dead cells. In the following step, cells were stained for surface markers with anti-mouse MHC-I-FITC (fluorescein isothiocyanate; clone 28.8.6; BD Biosciences) and anti-mouse PD-L1-PE (clone 10F.9G2; BioLegend). Isotype control antibodies for mouse IgG2a, κ (clone MOPC-173), and rat IgG2b, κ (clone RTK4530; BioLegend) were used for anti-MHC-I and anti-PD-L1 antibodies, respectively.

Cells isolated from tumors were also stained for viability as described above. Afterwards, the cells were treated with anti-mouse CD16/32 (Fc block, clone 93; BioLegend), and surface markers CD45, MHC-I, and PD-L1 were stained with the antibodies anti-CD45 (clone 30-F11, Alexa Fluor 700; BioLegend), anti-MHC-I, and anti-PD-L1, as described above. Data were analyzed with the FlowJo software, version 10.6 (BD Biosciences).

### 4.9. Cytokine Measurement

The presence of cytokines in tumors or in cell culture supernatants was evaluated by LEGENDplex assays (Mouse Inflammation Panel and Mouse Type 1/2 Interferon Panel; BioLegend) and the Invitrogen EGF Mouse ELISA Kit (Thermo Fisher Scientific) following the manufacturers’ protocols.

### 4.10. Statistical Analysis

The oncogenicity and tumor growth after immunotherapy were evaluated by two-way ANOVA and Bonferroni post-test. Tumor growth after neutralization of immune cells and results obtained by flow cytometry were analyzed by one-way ANOVA and Bonferroni post-test. A difference between groups was considered significant if *p* < 0.05. The calculations were performed using the Prism software, version 7 (Graph-Pad Software, San Diego, CA, USA).
